# *Cis*-Repression of *Foxq1* Expression Affects *Foxf2*-Mediated Gene Expression in Palate Development

**DOI:** 10.3389/fcell.2021.665109

**Published:** 2021-04-08

**Authors:** Jingyue Xu, Han Liu, Yu Lan, Rulang Jiang

**Affiliations:** ^1^Division of Developmental Biology, Cincinnati Children’s Hospital Medical Center, Cincinnati, OH, United States; ^2^Division of Plastic Surgery, Cincinnati Children’s Hospital Medical Center, Cincinnati, OH, United States; ^3^Departments of Pediatrics and Surgery, University of Cincinnati College of Medicine, Cincinnati, OH, United States; ^4^Shriners Hospitals for Children, Cincinnati, OH, United States

**Keywords:** bidirectional promoter, cleft palate, *cis* regulation, CRISPR, Forkhead, *Foxf2*, gene cluster, lincRNA

## Abstract

Disruption of *FOXF2*, encoding a member of the Forkhead family transcription factors, has been associated with cleft palate in humans and mice. *FOXF2* is located in a conserved gene cluster containing *FOXQ1*, *FOXF2*, and *FOXC1*. We found that expression of *Foxq1* is dramatically upregulated in the embryonic palatal mesenchyme in *Foxf2*^–/–^ mouse embryos. We show here that the *Foxf2* promoter-deletion mutation caused dramatically increased expression of the *cis*-linked *Foxq1* allele but had little effect on the *Foxq1* allele in *trans*. We analyzed effects of the *Foxf2* mutation on the expression of other neighboring genes and compared those effects with the chromatin domain structure and recently identified enhancer-promoter associations as well as H3K27ac ChIP-seq data. We show that the *Foxf2* mutation resulted in significantly increased expression of the *Foxq1* and *Exoc2* genes located in the same topologically associated domain with *Foxf2* but not the expression of the *Foxc1* and *Gmds* genes located in the adjacent chromatin domain. We inactivated the *Foxq1* gene in mice homozygous for a *Foxf2* conditional allele using CRISPR genome editing and generated *(Foxf2/Foxq1)^+/–^* mice with loss-of-function mutations in *Foxf2* and *Foxq1* in *cis*. Whereas the *(Foxf2/Foxq1)^–/–^* mice exhibited cleft palate at birth similar as in the *Foxf2*^–/–^ mice, systematic expression analyses of a large number of Foxf2-dependent genes revealed that the (*Foxf2*/*Foxq1)^–/–^* embryos exhibited distinct effects on the domain-specific expression of several important genes, including *Foxf1*, *Shox2*, and *Spon1*, in the developing palatal shelves compared with *Foxf2*^–/–^ embryos. These results identify a novel *cis*-regulatory effect of the *Foxf2* mutation and demonstrate that *cis-*regulation of *Foxq1* contributed to alterations in palatal gene expression in *Foxf2*^–/–^ embryos. These results have important implications for interpretation of results and mechanisms from studies of promoter- or gene-deletion alleles. In addition, the unique mouse lines generated in this study provide a valuable resource for understanding the cross-regulation and combinatorial functions of the *Foxf2* and *Foxq1* genes in development and disease.

## Introduction

The secondary palate separates the nasal cavity from the oral cavity and consists of the bony hard palate anteriorly and muscular soft palate posteriorly (Bush and Jiang, 2012). In mammals, the development of the secondary palate initiates as a pair of outgrowths from the oral side of embryonic maxillary processes, which grow vertically to form the palatal shelves flanking the developing tongue. As development proceeds, the palatal shelves reorient to the horizontal position above the tongue, grow toward and fuse with each other at the midline to form the roof of the oral cavity. Genetic or environmental perturbations of any of these developmental processes, including palatal shelf growth, elevation, and fusion, could cause cleft palate, one of the most common structural defects in humans (Chai and Maxson, 2006; Dixon et al., 2011; Bush and Jiang, 2012; Lan et al., 2015).

The development of secondary palate is regulated by a complex molecular network containing multiple signaling pathways and transcription factors. The Shh and Fgf signaling pathways have been shown to play a key role in regulating the palatal epithelial-mesenchymal interactions governing palatal shelf growth and patterning (Rice et al., 2004; Han et al., 2009; Lan and Jiang, 2009). Shh produced by the palatal epithelial cells signals to the palatal mesenchyme and forms a positive feedback loop with Fgf10 to coordinate cell proliferation in both the epithelium and mesenchyme during palate development (Rice et al., 2004; Lan and Jiang, 2009). In addition, Fgf7 produced by the palatal mesenchyme restricts the expression of *Shh* mRNAs to the oral side palatal epithelium to control the oral-nasal patterning (Han et al., 2009). Shh signaling pathway is also required to maintain the expression of the Forkhead genes *Foxf1* and *Foxf2* in the palatal mesenchyme (Lan and Jiang, 2009). We recently showed that a Shh-Foxf1/Foxf2-Fgf18-Shh molecular circuit regulates the proliferation of palatal mesenchymal cells during palatal shelf growth (Xu et al., 2016).

Foxf1 and Foxf2 are paralogous transcription factors of the Forkhead family with highly conserved amino acid sequences in the Forkhead DNA binding domain (Hellqvist et al., 1996). During palate development, *Foxf2* is expressed throughout the anterior-posterior axis of the palatal shelves while the expression of *Foxf1* is more restrict in the middle portion of palatal shelves (Nik et al., 2016; Xu et al., 2016). *Foxf2*^–/–^ mouse embryos display complete cleft secondary palate with multiple cellular defects. The proliferation of palatal mesenchymal cell is reduced, with the palatal shelf growth most significantly affected in the posterior portion in the *Foxf2*^–/–^ embryos (Nik et al., 2016; Xu et al., 2016). In addition, changes of extracellular matrix organization have also been shown to contribute to the cleft palate phenotype in *Foxf2*^–/–^ embryos (Nik et al., 2016; Xu et al., 2020). Mutations in *FOXF2* have been associated with cleft palate in humans (Jochumsen et al., 2008; Bu et al., 2015). A recent study reported a familial palate defect with absent uvula, short posterior border of the soft palate, and abnormal tonsillar pillar (Seselgyte et al., 2019). Further genetic studies identified a missense variant in *FOXF2* as the likely cause of this condition (Seselgyte et al., 2019). Thus, better understanding of the molecular mechanisms mediating Foxf2 function in palate development in mice will improve our understanding of cleft palate pathogenesis in humans.

To investigate the molecular mechanisms mediating Foxf2 function in palate development, we have used a combination of whole transcriptome RNA sequencing (RNA-seq) and chromatin immunoprecipitation-sequencing (ChIP-seq) mediated genome wide mapping of Foxf2 binding sites to identify direct Foxf2 target genes in the developing palatal mesenchyme (Xu et al., 2016, 2020). In addition to identifying *Fgf18* as a direct Foxf2 target gene that acts in the Shh-Foxf2-Fgf18-Shh molecular circuit to control palatal shelf growth (Xu et al., 2016), we showed that a number of genes encoding components of the extracellular matrix and a group of genes encoding transcription factors are direct Foxf2 target genes in the developing palatal mesenchyme cells (Xu et al., 2020). Among these, *Foxq1*, which encodes a Forkhead transcription factor with high amino acid sequence similarity with the Foxf2 protein, is one of the most significantly up-regulated genes in the developing palatal mesenchyme in *Foxf2*^–/–^ embryos (Xu et al., 2020). *Foxq1* is expressed at very low levels in the palatal mesenchyme in wildtype mouse embryos (Xu et al., 2020). *Foxq1*^–/–^ mutant mice exhibited defects in hair follicle development and gastric acid secretion but no palatal defect has been reported (Hong et al., 2001; Goering et al., 2008). Remarkably, *Foxq1* and *Foxf2* are closely linked genes in an evolutionarily conserved gene cluster in all vertebrate genomes (Wotton and Shimeld, 2006). In both human and mouse genomes, the *Foxq1* gene is located directly upstream of *Foxf2*. Previous studies using breast cancer cell lines have suggested that FOXF2 and FOXQ1 have opposite functions in regulating epithelial-mesenchymal transition and that they repress the expression of each other (Zhang et al., 2011; Wang et al., 2015; Kang et al., 2019). Thus, the significant upregulation of *Foxq1* expression in the developing palatal mesenchyme in *Foxf2*^–/–^ embryos suggest that Foxf2-mediated regulation of *Foxq1* expression may play an important role in palatogenesis. To address this possibility and gain better understanding of Foxf2-mediated regulation of *Foxq1* expression during palate development, we have generated mice carrying mutations in both *Foxf2* and *Foxq1* in *cis* using CRISPR/Cas9 mediated genome editing (Cong et al., 2013; Wang et al., 2013) and our results identify an unexpected *cis*-regulation of *Foxq1* by *Foxf2* in the developing palate.

## Materials and Methods

### Mice

*Foxf2*^*tm1Rhc*^ (*Foxf2*^*c/c*^) and *Foxf2*^+/–^ mice have been described previously (Hoggatt et al., 2013; Bolte et al., 2015) and were maintained by intercrossing or by crossing with C57BL/6J inbred mice. To generate *Foxf2*^*c/c*^; *Foxq1*^+/–^ mice, two synthetic guide RNAs (sgRNAs) (50 ng/μl each) targeting the genomic sequence flanking the Forkhead domain-coding sequence in the *Foxq1* gene were co-injected with humanized *Cas9* mRNAs (50 ng/μl) into zygotes of *Foxf2*^*c/c*^ mice (Figure 2A). The target sequences of the two sgRNAs are: 5′-GCAGCAAGCCGTACACGCGG-3′ and 5′-GCGAATACACCTTCGCCGAC-3′. Injected eggs were transferred on the same day into the oviductal ampulla of pseudopregnant CD-1 female mice at approximately 25 eggs per recipient. *Foxq1* gene-modified founder mice were identified by PCR assay and then the exact nucleotide changes at the edited *Foxq1* locus were determined by Sanger sequencing. Mice carrying two independent *Foxq1*-deletion alleles, lacking 509 bp and 494 bp, respectively, of the *Foxq1* coding region (*Foxq1*^*D509*^ and *Foxq1*^*D494*^) were used in this study. Founder mice were crossed to *Foxf2*^*c/c*^ mice to generate the *Foxf2*^*c/c*^; *Foxq1*^+/–^ mice. Genotypically verified G1 *Foxf2*^*c/c*^; *Foxq1*^+/–^ mice were intercrossed to generate *Foxf2*^*c/c*^; *Foxq1*^–/–^ homozygotes. In addition, *Foxf2*^*c/c*^; *Foxq1*^+/–^ mice were crossed to *EIIa-Cre* transgenic mice (Lakso et al., 1996) to inactivate the *Foxf2*^*c*^ allele and generate the *(Foxf2/Foxq1)^+/–^* mice. *(Foxf2/Foxq1)^+/–^* mice were intercrossed to generate *(Foxf2/Foxq1)^–/–^* homozygous embryos for analyses. For timed mating, noon of the day on which a vaginal plug was identified was designated as embryonic day (E) 0.5. All animal work procedures were performed following recommendations in the Guide for Care and Use of Laboratory Animals by the National Institutes of Health and approved by the Institutional Animal Care and Use Committee (IACUC) at Cincinnati Children’s Hospital Medical Center. This study conformed with the ARRIVE (Animal Research: Reporting of *in vivo* Experiments) guidelines for preclinical animal studies.

### Histology, *in situ* Hybridization, and Immunofluorescent Staining

Embryos were collected and processed for histology, immunostaining, or *in situ* hybridization as described previously (Xu et al., 2016). For histology and immunofluorescent staining, the embryos were fixed in 4% paraformaldehyde (PFA), dehydrated through an ethanol series, embedded in paraffin, and sectioned at 7 μm thickness. The goat anti-Foxf1 (AF4798; R&D) antibody was used to detect the Foxf1 protein. Images were taken using a Nikon DS-Qi2 microscope (Nikon Instruments Inc., Melville, NY, United States).

### RNA Extraction, Real-Time Polymerase Chain Reaction (RT-qPCR), and Enzyme Digestion Assay

Whole palatal shelves of E13.5 embryos were manually dissected in ice-cold phosphate buffered saline. Total RNAs were extracted using the RNeasy micro kit (74004; Qiagen Inc., Germantown, Maryland). First-strand cDNAs were prepared using the SuperScript III First-Strand Synthesis System (18080-051; Invitrogen, Carlsbad, California), and real-time qPCR was performed using a CFX96 Real-Time System (Bio-Rad, Hercules, California) with conditions recommended by the manufacturer. Relative levels of mRNAs in each sample were normalized to that of *Hprt* mRNAs. For restriction enzyme digestion assay to measure allele specific expression of *Foxq1* mRNAs, a pair of primers was designed to flank a single nucleotide polymorphism in the 3′ untranslated region (UTR) of the *Foxq1* gene (rs29587452) between the C57BL/6J and 129X1/SvJ mouse strains. Purified PCR products amplified from wildtype, *Foxf2^+/–^*, and *Foxf2*^–/–^ cDNA samples were digested overnight using *Aci*I (NEB, R0551L) at 37^*o*^C. Quantification of the digested and undigested DNA fragments was performed by QIAxcel Advanced using QIAxcel^®^ ScreenGel software (QIAGEN, Cat# 9021163).

For statistical analysis, all results were presented as mean ± SEM. Student’s *t* test was used for pairwise comparison. One-way ANOVA followed by Newman–Keuls *post hoc* test was used to compare all pairs when more than two genotypes were included. *P* < 0.05 was considered significantly different.

### Genomic Data Retrieval and Analysis

The whole genome chromosome conformation capture (Hi-C) data and topologically associated domain (TAD) map of mouse embryonic stem cells were retrieved from the 3D Genome Browser^1^ (Wang et al., 2018). The original Hi-C data was from Bonev_2017- raw (Bonev et al., 2017; Wang et al., 2018), and assembled into mm10 reference genome with the resolution set at 10 kb. The histone H3K27ac ChIP-seq data and the bigwig file of the E12.5 mouse embryonic posterior palatal shelves was obtained from the NCBI GEO database^2^ (accession number GSE138721) (Xu et al., 2019). The replicated associations between enhancers and gene promoter data were retrieved from the UCSC Genome Browser^3^ (Gorkin et al., 2020).

## Results

### Disruption of *Foxf2* Causes Significantly Increased Expression of the Linked *Foxq1* Allele in *Cis* in the Developing Palate

**FIGURE 1:**
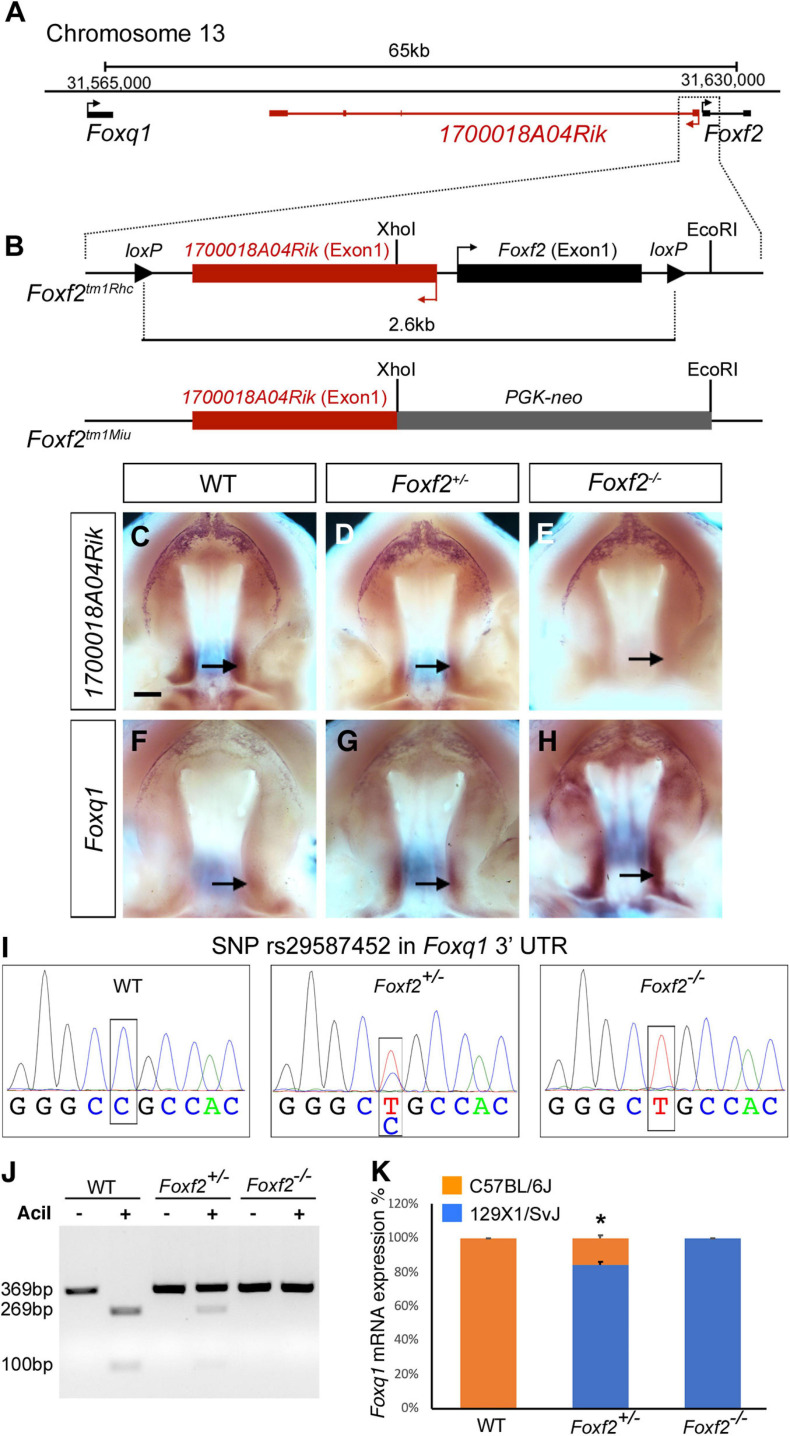
Disruption of *Foxf2* causes increased expression of the linked *Foxq1* allele in *cis* in the developing palate. **(A)** Schematics of the *Foxq1*-*Foxf2* gene locus. *Foxq1* is located 65 kb upstream of *Foxf2*, and the two genes are transcribed from the same strand. The lncRNA gene *1700018A04Rik* is located between *Foxq1* and *Foxf2*, and is transcribed from the opposite DNA strand. **(B)** Schematics of the gene targeting strategies of two *Foxf2* mutant mouse lines, *Foxf2*^*tm1Rhc*^ and *Foxf2*^*tm1Miu*^. Note that both *Foxf2* gene-knockout alleles inactivated both *Foxf2* and *1700018A04Rik*. **(C–H)** Comparison of patterns of expression of *1700018A04Rik* **(C–E)** and *Foxq1* **(F–H)** mRNAs in the E13.5 wild-type (WT) **(C,F),** *Foxf2*^+/–^ **(D,G)**, and *Foxf2*^–/–^ **(E,H)** embryos. Note that *1700018A04Rik* mRNAs were downregulated, while *Foxq1* mRNAs were upregulated along the anterior-posterior axis, and more significantly increased in the posterior (arrows) subdomains of *Foxf2*^+/–^ and *Foxf2*^–/–^ mutant palatal shelves. Scale bar, 400 μm. **(I)** Sanger sequencing verification of a SNP (rs29587452) in the *Foxq1* 3′ UTR that could distinguish the mRNAs transcribed from the *Foxf2* mutation-linked *cis Foxq1* allele (129SvEv background) versus the mRNAs transcribed from the *Foxq1* allele linked to the wildtype *Foxf2* locus (C57BL/6J background) in heterozygous embryos. **(J)** DNA electrophoresis gel image showing restriction length polymorphism of RT-PCR products from the 3′ UTR of the *Foxq1* mRNAs from E13.5 WT and *Foxf2*^+/–^ embryonic palatal tissues. **(K)** Quantification of the ratio of *Aci*I-digested and undigested DNA fragments by QIAxcel advanced system. Note that over 80% of the *Foxq1* mRNAs in the *Foxf2*^+/–^ palatal shelves was expressed from the *Foxf2* mutation-linked cis *Foxq1* allele (middle column) (*n* = 5). *p < 0.05.

Both *Foxf2* and *Foxq1* are located on mouse Chromosome 13, with *Foxq1* at about 65 kb proximal and upstream of *Foxf2*, and with the two genes in the same transcriptional orientation (Figure 1A). In between *Foxq1* and *Foxf2*, there is an uncharacterized long non-coding RNA (lncRNA) gene, named *1700018A04Rik*, which is transcribed from the opposite DNA strand with the most 5′ transcription start site (TSS) located only about 300 bp from the TSS of *Foxf2* (Figures 1A,B). To date, three *Foxf2* gene-targeted mouse lines have been reported (Wang et al., 2003; Hoggatt et al., 2013; Bolte et al., 2015; Reyahi et al., 2015). In *Foxf2^*tm1Rhc*^*, the *Foxf2* conditional allele that we are using, the two *loxP* sites flank a genomic region containing both Exon-1 of the *Foxf2* gene and Exon-1 of the *1700018A04Rik* gene (Figure 1B; Hoggatt et al., 2013; Bolte et al., 2015). Cre mediated deletion of the floxed region in this allele deletes the Exon-1 and the promoter of both genes. In another *Foxf2* conditional allele, *Foxf2*^*tm1Pca*^, the two *loxP* sites also flank a genomic region containing the promoter and Exon-1 of the *Foxf2* gene (Reyahi et al., 2015), whereas a *Foxf2* conventional knockout allele, *Foxf2*^*tm1Miu*^, was generated by replacing the *Xho*I-*Eco*RI genomic region containing the promoter and Exon-1 region of the *Foxf2* gene with a *PGK-Neo* expression cassette (Wang et al., 2003). Thus, all three *Foxf2* gene-knockout alleles reported to date likely inactivated both *Foxf2* and *1700018A04Rik*. *In situ* hybridization analysis showed that *1700018A04Rik* mRNAs were expressed in the posterior region of the developing palatal shelves in wildtype embryos (Figure 1C), which overlaps with the posterior palate domain of strong *Foxf2* expression as reported previously (Xu et al., 2016). No *1700018A04Rik* mRNA expression was detected in the *Foxf2*^–/–^ mutant embryos whereas *Foxf2*^+/–^ embryos showed significantly reduced *1700018A04Rik* mRNA expression in comparison with the wildtype littermates (Figures 1C–E). Analysis of *Foxq1* mRNA expression revealed an almost mirror image pattern, with wildtype embryos exhibiting very low level of *Foxq1* mRNA expression in the posterior palatal shelves and *Foxf2*^+/–^ embryos exhibiting dramatically increased *Foxq1* mRNA expression while *Foxf2*^–/–^ mutant embryos exhibiting even stronger *Foxq1* mRNA expression that expanded from the posterior domain to the anterior regions of the palatal shelves (Figures 1F–H).

The expression pattern of *Foxq1* in the *Foxf2*^–/–^ mutant palatal shelves resembles the expression pattern of *Foxf2* in wildtype embryos (Xu et al., 2016). The physical linkage of *Foxq1* with *Foxf2* in the genome and the dose-dependent alteration of *Foxq1* expression in the *Foxf2*^+/–^ and *Foxf2*^–/–^ embryos raise the question whether the *Foxf2* gene disruption directly affects the expression of the linked *Foxq1* allele in *cis*. Since the *Foxf2*^*tm1Rhc*^ allele was generated by homologous recombination based gene targeting in mouse embryonic stem cells of the C57BL/6 × 129SvEv hybrid genetic background (Hoggatt et al., 2013; Bolte et al., 2015), we first examined whether a single nucleotide polymorphism (SNP) (rs29587452) in the *Foxq1* 3′ UTR between C57BL/6J and 129X1/SvJ mouse strains^4^ could distinguish the mRNAs transcribed from the *Foxf2* mutation-linked *cis Foxq1* allele versus the mRNAs transcribed from the *Foxq1* allele linked to the wildtype *Foxf2* locus in heterozygous embryos. Sequencing analysis of RT-PCR products from wildtype, *Foxf2*^+/–^, and *Foxf2*^–/–^ embryos showed that the *Foxq1* allele *cis*-linked with the *Foxf2* mutation was of the 129X1/SvJ genotype and distinct from the C57BL/6J allele for rs29587452 (Figure 1I), indicating that the targeted *Foxf2*^*tm1Rhc*^ allele was of 129SvEv origin and that the *Foxq1* allele in the 129SvEv background shares the same variant in the 3′ UTR as in the 129X1/SvJ background. The C57BL/6J sequence at the SNP site contains a recognition sequence for the *Aci*I endonuclease (CCGC) that is disrupted in the 129SvEv allele. We designed a pairs of PCR primers flanking SNP rs29587452 and used *Aci*I restriction fragment polymorphism to analyze possible differential expression of the two *Foxq1* alleles in the *Foxf2*^+/–^ mutant palate shelves (Figure 1J). We found that over 80% of the *Foxq1* mRNAs in the *Foxf2*^+/–^ palatal shelves was expressed from the *Foxf2* mutation-linked *cis Foxq1* allele (Figure 1K), suggesting that the increased *Foxq1* mRNA expression in the *Foxf2*^+/–^ and *Foxf2*^–/–^ embryos resulted primarily from a *cis*-regulatory effect of the *Foxf2* mutation.

### Generation of *Foxf2/Foxq1* Double Mutant Mice and Validation of *Cis*-Regulation of *Foxq1* Expression by *Foxf2* Disruption

**FIGURE 2:**
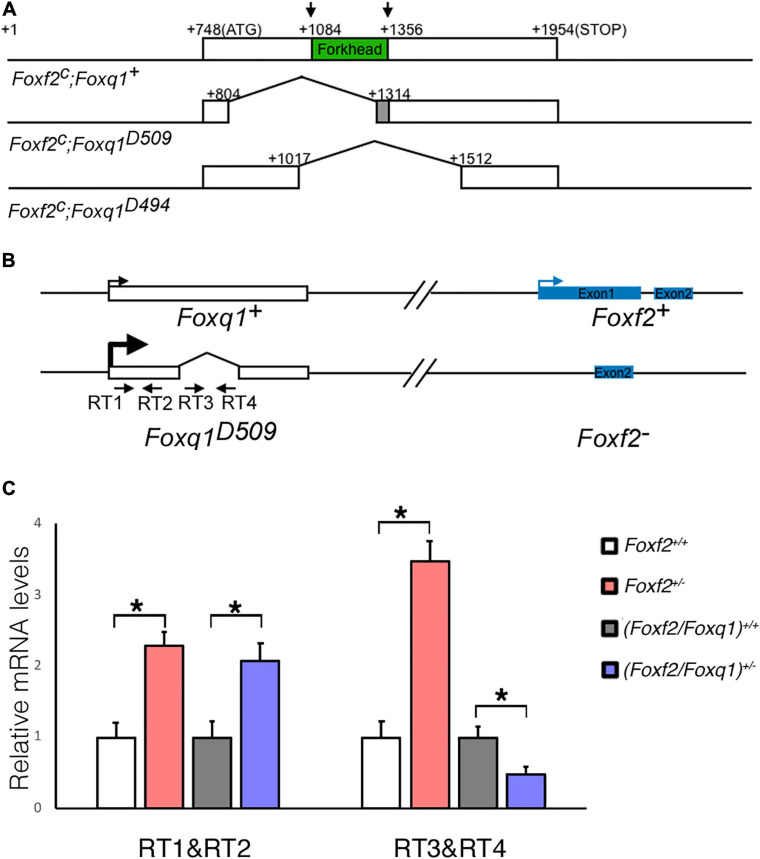
Generation of *Foxf2/Foxq1* double mutant mouse. **(A)** Schematics of the strategy for generating *Foxf2*^*c*^; *Foxq1*^–^ mice using the CRISPR/cas9 approach. The top row shows the genomic organization of the mouse *Foxq1* locus. The coding sequence is boxed with the Forkhead-domain coding region filled in green. The two vertical arrows indicate the positions of recognition sites of the two sgRNAs used for CRISPR genome editing. The second and third rows show the two edited *Foxf2*^*c*^; *Foxq1*^–^ mouse alleles, *Foxf2*^*c*^; *Foxq1^*D509*^*, and *Foxf2*^*c*^; *Foxq1*^*D494*^, respectively. **(B)** Schematics of the strategy for investigating the allele-specific effects of the *Foxf2* mutation on the linked *Foxq1* gene. A pair of primers (RT1&RT2) was designed to detect the *Foxq1* mRNAs expressed from both alleles. Another pair of primers (RT3&RT4) was designed to detect the *Foxq1* mRNAs expressed from only the wildtype *Foxq1* allele. **(C)** Real time RT-qPCR analysis of the levels of expression of *Foxq1* mRNAs in E13.5 palatal shelves in *Foxf2*^+/+^, *Foxf2*^+/–^,*(Foxf2/Foxq1)^+/+^*, and *(Foxf2/Foxq1)^+/–^* embryos (*n* ≥ 4). *p < 0.05.

To further investigate the regulation and function of *Foxq1* in the *Foxf2* mutant mice, we used the CRISPR/Cas9 genome editing technology to inactivate the *Foxq1* gene in the *Foxf2*^*c/c*^ mice. Two independent mouse lines, carrying a deletion of 509 (*Foxf2*^*c*^; *Foxq1*^*D509*^) and 494 (*Foxf2*^*c*^; *Foxq1*^*D494*^) bp, respectively, spanning the entire Forkhead domain-coding region of the *Foxq1* gene were established and used in this study (Figure 2A). Both *Foxq1*^*D509*^ and *Foxq1*^*D494*^ alleles resulted in identical silky coat phenotypes in the homozygous mutants (Supplementary Figure 1), which are similar to the previously reported phenotype of *Foxq1* null mutant mice (Hong et al., 2001; Goering et al., 2008). Thus, we refer to these alleles as *Foxq1*^–^. We crossed the *Foxf2*^*c/c*^; *Foxq1*^+/–^ mice with the *EIIa-Cre* transgenic mice (Lakso et al., 1996) to delete the floxed *Foxf2* region in the early embryo and subsequently crossed the (*Foxf2/Foxq1*)*^+/–^* progeny to C57BL/6J mice to establish the *(Foxf2/Foxq1)^+/–^* mouse colony. These *(Foxf2/Foxq1)^+/–^* mice allowed us to further investigate allele-specific effects of the *Foxf2* mutation on the linked *Foxq1* allele by direct quantitative comparison of allele-specific *Foxq1* expression between different embryos of distinct genotypes (Figure 2B). As shown in Figures 2B,C, we found that the total amount of mRNAs from the two *Foxq1* alleles expressed in the E13.5 palatal shelves were increased by more than twofold in both *Foxf2*^+/–^ and *(Foxf2/Foxq1)^+/–^* embryos in comparison with their wildtype littermates. However, the amount of mRNAs produced from the wildtype *Foxq1* allele in the *(Foxf2/Foxq1)^+/–^* embryos was only about 50% of the amount of *Foxq1* mRNAs in the wildtype sample (Figures 2B,C). Together with the result that the *Foxq1* mRNAs in the palatal mesenchyme in the *Foxf2*^+/–^ embryos was predominantly produced by the *Foxf2* mutation-linked *cis Foxq1* allele (Figures 1J,K), these results indicate that the *Foxf2* mutation caused the significantly increased expression of the *cis*-linked *Foxq1* allele but had little effect on the *Foxq1* allele in *trans*.

### The *Cis*-Regulatory Effect of the *Foxf2* Mutation on *Foxq1* Expression Is Highly Specific and Correlates With Local Genome Organization

**FIGURE 3:**
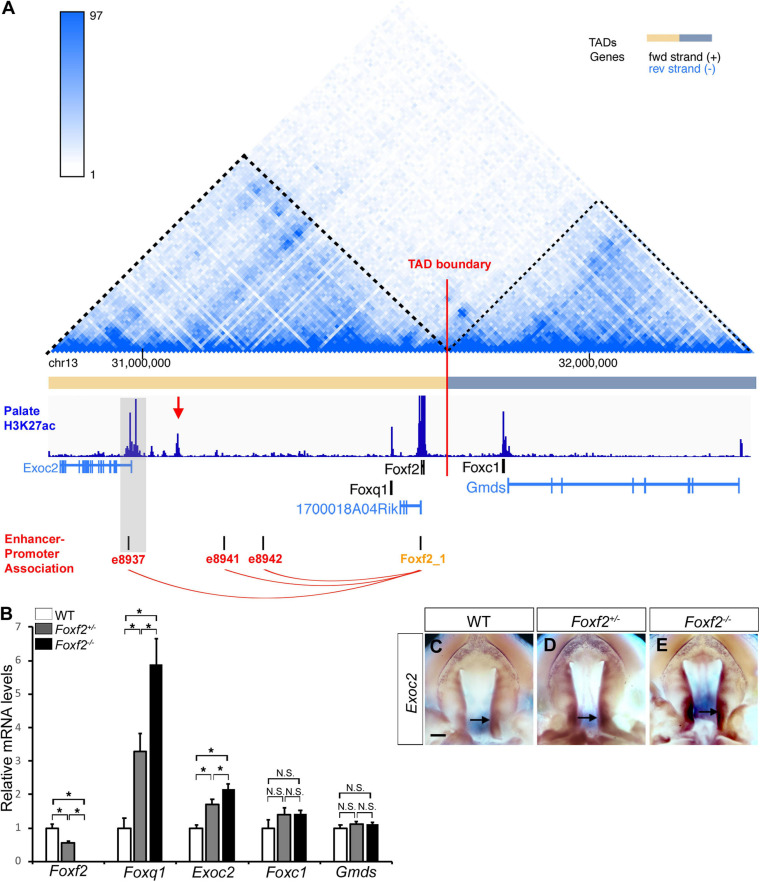
Analysis of genome organization around the *Foxf2*-*Foxq1* gene cluster and the effect of the *Foxf2* mutation on expression of neighboring genes in palate development. **(A)** Hi-C map and histone H3K27ac ChIP-seq peaks in the genomic region containing the *Exoc2*, *Foxq1*, *1700018A04Rik*, *Foxf2*, *Foxc1*, and *Gmds* genes (Chr13: 30,790,000-32,370,000). The top panel shows the chromatin interaction frequency heatmap from Hi-C analysis of mouse embryonic stem cells (3dgenome.fsm.northwestern.edu) (Bonev et al., 2017; Wang et al., 2018). The color of the heatmap indicates the level of normalized interaction. The identified topological association domains (TADs) are indicated by orange and gray color, respectively. The middle panel shows histone H3K27ac ChIP-seq peak signal plot in deep blue color [data from Xu et al. (2019)]. A red arrow points to a highly enriched H3K27ac peak in the intergenic region between *Exoc2* and *Foxq1*. Transcription orientation of the genes is indicated by black (“ + ” strand) or blue color (“-” strand). The bottom panel shows the replicated associations between enhancers and the *Foxf2* gene promoter, retrieved from ENCODE3 and EPDnew (https://genome.ucsc.edu). Three enhancers (e8937, e8941 and e8942) were annotated to show replicated associations with the *Foxf2* promoter in mouse fetal tissues. Note that enhancer e8937 is located in intron-1 of the *Exoc2* gene and colocalized with a H3K27ac peak from the E12.5 mouse posterior palatal tissues (highlighted in gray). **(B)** Real time RT-qPCR analysis of the levels of expression of *Foxf2, Foxq1, Exoc2, Foxc1*, and *Gmds* mRNAs in the E13.5 palatal shelves in wildtype (WT), *Foxf2*^+/–^ and *Foxf2*^–/–^ embryos (*n* ≥ 4). *p < 0.05. N.S., not significantly different. **(C–E)** Palatal view of whole mount embryonic upper jaws showing *Exoc2* mRNA expression in the palatal tissues in E13.5 wildtype (WT) **(C)**, *Foxf2*^+/–^ **(D)**, and *Foxf2*^–/–^ **(E)** embryos. Arrow points to posterior region of the palatal shelves. Scale bar, 400 μm.

Genome-wide chromosome conformation capture and sequencing (Hi-C) studies have demonstrated that mammalian genomes are organized into a series of topologically associated domains (TADs), megabase-scale genomic intervals where interactions between enhancers and gene promoters take place more frequently within than across adjacent TADs (Dixon et al., 2012; Nora et al., 2012; Bonev et al., 2017). Although the *Foxc1* gene is located immediately downstream of the *Foxf2* gene in the evolutionarily conserved *Foxq1*-*Foxf2*-*Foxc1* gene cluster, analysis of previously generated Hi-C data from mouse and human embryonic stem cells showed that the *Foxq1* and *Foxf2* genes are located in the same TAD while the *Foxc1* gene is located in a separate adjacent TAD (Figure 3A; Dixon et al., 2012; Haliburton et al., 2016). RT-qPCR analysis showed that, in contrast to significantly increased expression of *Foxq1* in E13.5 *Foxf2*^+/–^ and *Foxf2*^–/–^ palatal tissues, the levels of *Foxc1* mRNA expression was not significantly altered in *Foxf2*^+/–^ and *Foxf2*^–/–^ palatal tissues in comparison with the wildtype littermates (Figure 3B). Expression of *Gmds*, which is located downstream of but in the same TAD with *Foxc1*, was not significantly altered either (Figure 3B). On the other hand, expression of *Exoc2*, a gene located about 600 kb upstream of *Foxf2*, was significantly increased in the palatal tissues in *Foxf2*^+/–^ embryos and further increased in *Foxf2*^–/–^ embryos in comparison with wildtype littermates (Figure 3B). *In situ* hybridization analysis showed that *Exoc2* mRNA expression was increased throughout the anterior-posterior axis of the palatal shelves, with particularly strong upregulation in the posterior region of the palatal shelves in the *Foxf2*^+/–^ and *Foxf2*^–/–^ embryos (Figures 3C–E). Analysis of the Hi-C data (Bonev et al., 2017) indicated that *Exoc2* is located in the same TAD with *Foxf2* and *Foxq1* (Figure 3A). Furthermore, recent analysis of data from systematic epigenomic and transcriptome profiling of mouse embryonic tissues at multiple developmental stages in the Encyclopedia of DNA Elements (ENCODE) project identified many thousands of enhancer-promoter interactions, among which three long distance enhancers located between *Exoc2* and *Foxq1* exhibited strong replicated associations with the *Foxf2* gene promoter but not with the *Foxq1* gene promoter (Gorkin et al., 2020; Figure 3A). Remarkably, analysis of recently generated ChIP-seq data for histone H3K27 acetylation chromatin marks in the posterior palatal shelves of E12.5 mouse embryos (Xu et al., 2019) showed that one prominent H3K27ac peak colocalized with one of the distal enhancers, e8937 located in intron-1 of the *Exoc2* gene, that showed strong replicated association with the *Foxf2* gene promoter in multiple embryonic tissues (Gorkin et al., 2020; Figure 3A). In addition, analysis of the H3K27ac ChIP-seq data (Xu et al., 2019) revealed another strong peak in the intergenic region between *Exoc2* and *Foxq1*, which likely marks an active enhancer in the E12.5 mouse palatal tissues (Figure 3A). Together, these data suggest that expression of *Foxf2* in the palatal mesenchyme in wildtype embryos is controlled by distant enhancers located close to the *Exoc2* gene and deletion of the *Foxf2* gene promoter likely resulted in increased activation of the nearby *Foxq1* and *Exoc2* genes within the same TAD in the *Foxf2*^+/–^ and *Foxf2*^–/–^ palatal mesenchyme by those enhancers.

### Analysis of the Function of *Foxq1* in Foxf2-Mediated Regulation of Palate Development

**FIGURE 4:**
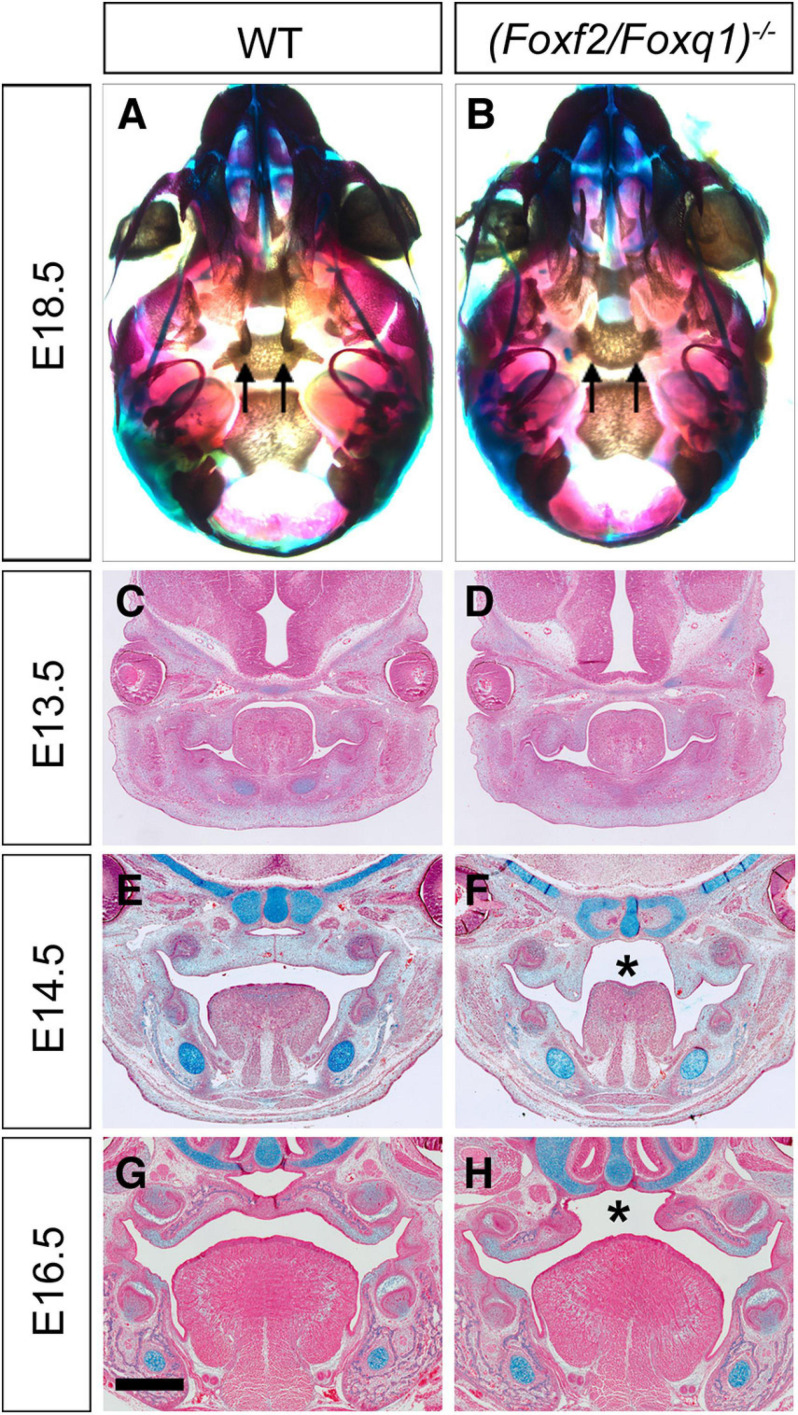
Phenotypical analysis of palate developmental defects in *(Foxf2/Foxq1)^–/–^* mutant mouse embryos. **(A)** Skeletal preparations of heads of E18.5 wildtype (WT) **(A)** and *(Foxf2/Foxq1)^–/–^* **(B)** embryos. Note that the pterygoid processes (arrows) were extremely hypoplastic in *(Foxf2/Foxq1)^–/–^* **(B)** compare with WT **(A)** embryos. **(C–H)** Representative frontal sections of wildtype **(C,E,G)** and *(Foxf2/Foxq1)^–/–^* **(D,F,H)** embryos at E13.5 **(C,D)**, E14.5 **(E,F)**, and E16.5 **(G,H)**. Note that *(Foxf2/Foxq1)^–/–^* embryos displayed cleft palate (“*” in F and H marks the gap between the bilateral palatal shelves). Scale bar, 500 μm.

Intercrossing of *(Foxf2/Foxq1)^+/–^* mice generated *(Foxf2/Foxq1)^–/–^* mutant mice with over 80% of the homozygous mutants exhibited complete cleft palate (Figures 4A,B). Analysis of the *(Foxf2/Foxq1)^–/–^* embryos at multiple developmental stages by histology or skeletal preparations revealed that craniofacial anomalies, including cleft palate, were similar as the phenotypes of *Foxf2*^–/–^ embryos described previously (Xu et al., 2016). Similar with *Foxf2*^–/–^ mutant embryos, *(Foxf2/Foxq1)^–/–^* embryos displayed defects in palatal shelf elevation (Figures 4C–H) and malformed pterygoid processes (Figures 4A,B).

**FIGURE 5:**
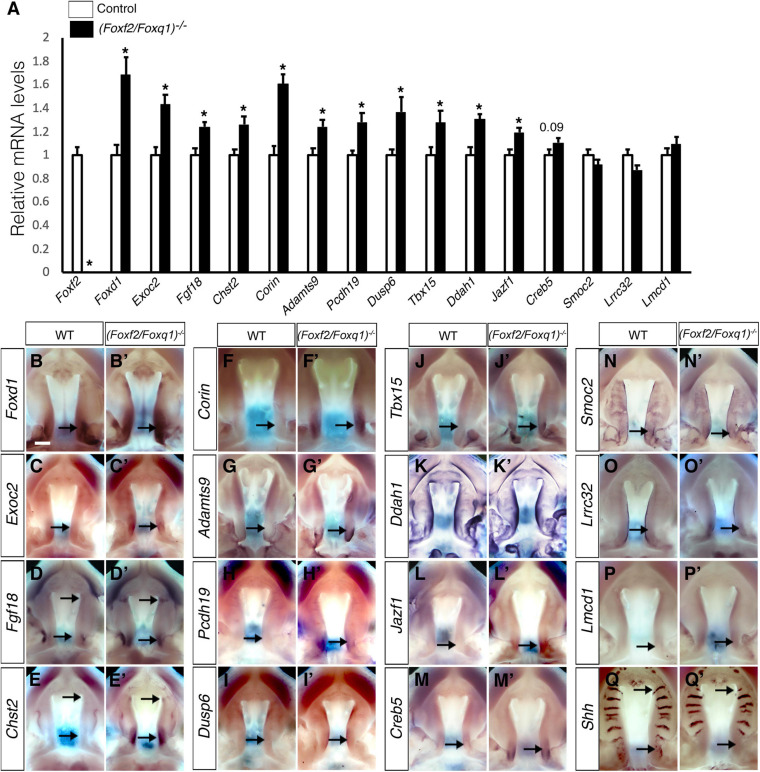
Analysis of expression of Foxf2 target genes in the developing palatal shelves. **(A)** Real time RT-qPCR analysis of the levels of expression of *Foxf2*, *Foxd1*, *Exoc2*, *Fgf18*, *Chst2*, *Corin*, *Adamts9*, *Pcdh19*, *Dusp6*, *Tbx15*, *Ddah1*, *Jazf1*, *Creb5*, *Smoc2*, *Lrrc32*, and *Lmcd1* mRNAs in the E13.5 palatal shelves in control and *(Foxf2/Foxq1)^–/–^* embryos (*n* = 7). *p < 0.05. **(B–Q, B′–Q′)** Comparison of patterns of expression of *Foxd1*, *Exoc2, Fgf18, Chst2, Corin, Adamts9, Pcdh19, Dusp6, Tbx15, Ddah1, Jazf1, Creb5, Smoc2, Lrrc32, Lmcd1*, and *Shh* mRNAs in E13.5 wild-type (WT) **(B–Q)** and *(Foxf2/Foxq1)^–/–^* **(B′–Q′)** mutant embryos. Scale bar, 400 μm.

**FIGURE 6:**
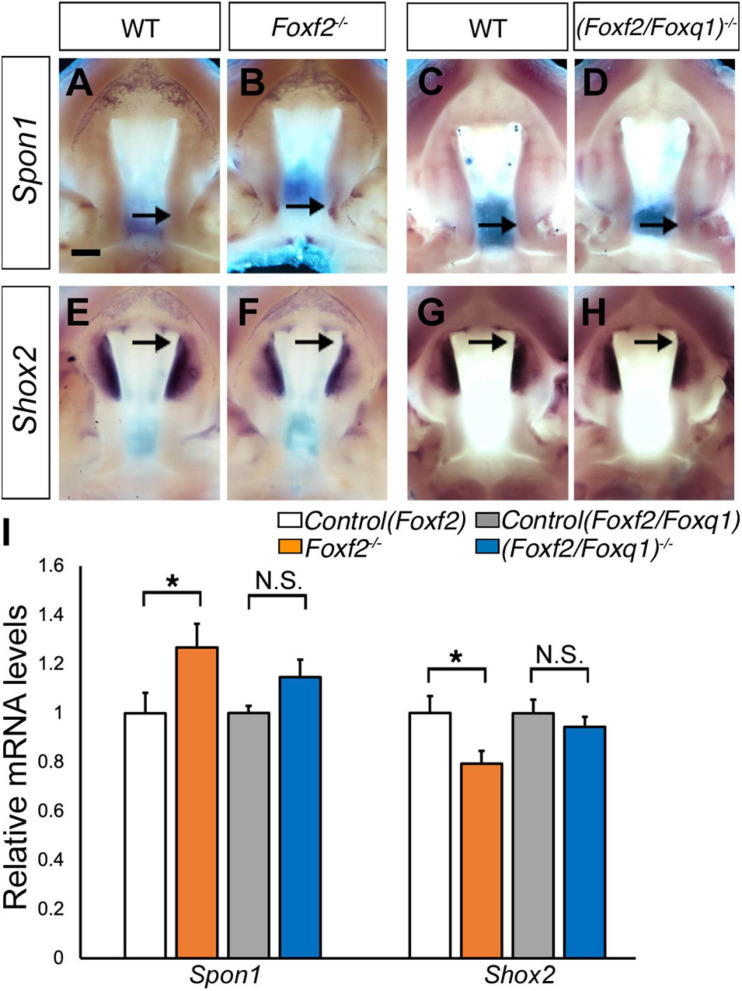
Analysis of expression of *Spon1* and *Shox2* in the developing palatal shelves. **(A–H)** Comparison of patterns of expression of *Spon1* **(A–D)** and *Shox2* **(E–H)** mRNAs in E13.5 wild-type (WT) **(A,C,E,G)**, *Foxf2*^–/–^ **(B,F)** and *(Foxf2/Foxq1)^–/–^* **(D,H)** mutant embryos. The expression of *Spon1* was increased in a specific domain in posterior palatal shelves in *Foxf2*^–/–^ embryos [arrow in **(B)**]. The expression of *Shox2* was decreased in the anterior palatal shelves in *Foxf2*^–/–^ embryos (arrow in **F**). Scale bar, 400 μm. **(I)** Real time RT-qPCR analysis of the levels of expression of *Spon1* and *Shox2* mRNAs in E13.5 palatal shelves in the control, *Foxf2*^–/–^ and *(Foxf2/Foxq1)^–/–^* embryos (*n* = 7) *p < 0.05. N.S., not significantly different.

We then investigated whether the increased expression of *Foxq1* contributed to changes in gene expression as previously reported in *Foxf2*^–/–^ embryos. We examined both levels and patterns of expression of multiple previously identified differentially expressed genes between the *Foxf2*^–/–^ and control embryos. For most of these genes, the differential expression changes were similarly observed in (*Foxf2/Foxq1)^–/–^* embryos as previously reported for *Foxf2*^–/–^ mutant embryos (Xu et al., 2020; Figure 5). The expression of *Foxd1*, *Exoc2*, *Fgf18*, *Chst2*, *Corin*, *Adamts9*, *Pcdh19*, *Dusp6*, *Tbx15*, *Jazf1*, *Creb5*, *Smoc2*, *Lrrc32*, and *Lmcd1* were increased in the posterior palatal shelves in (*Foxf2/Foxq1)^–/–^* embryos in comparison with wildtype littermates at E13.5 (Figure 5). The expression of *Shh* mRNAs was down-regulated in the posterior palate as well as in the anterior domain corresponding to the most anterior palatal rugae (Figures 5Q,Q′), which is also similar to the pattern of *Shh* expression in *Foxf2*^–/–^ embryos (Xu et al., 2016). Nevertheless, we found that expression of *Spon1*, which was significantly increased in the posterior palatal shelves in *Foxf2*^–/–^ mutant embryos at E13.5 (Figures 6A,B,I) but was not significantly increased in the E13.5 palatal shelves in *(Foxf2/Foxq1)^–/–^* embryos compared with the wildtype littermates (Figures 6C,D,I). In addition, while *Shox2* was strongly expressed throughout the anterior half of the palatal shelves in wildtype embryos (Figures 6E,G) and was significantly decreased in the most anterior region of the *Foxf2*^–/–^ mutant palatal shelves at E13.5 (Figures 6F,I), *Shox2* expression in the anterior region of the palatal shelves appeared partly restored in the *(Foxf2/Foxq1)^–/–^* mutant embryos (Figures 6H,I). These results indicate that the increased expression of *Foxq1* affected expression of some previously identified Foxf2-dependent gene expression patterns in both the anterior and posterior regions of the palatal shelves in the *Foxf2*^–/–^ embryos.

### Foxq1 Partly Complemented Foxf2-Mdiated Regulation of *Foxf1* Expression in the Developing Palatal Shelves in *Foxf2*^–/–^ Embryos

**FIGURE 7:**
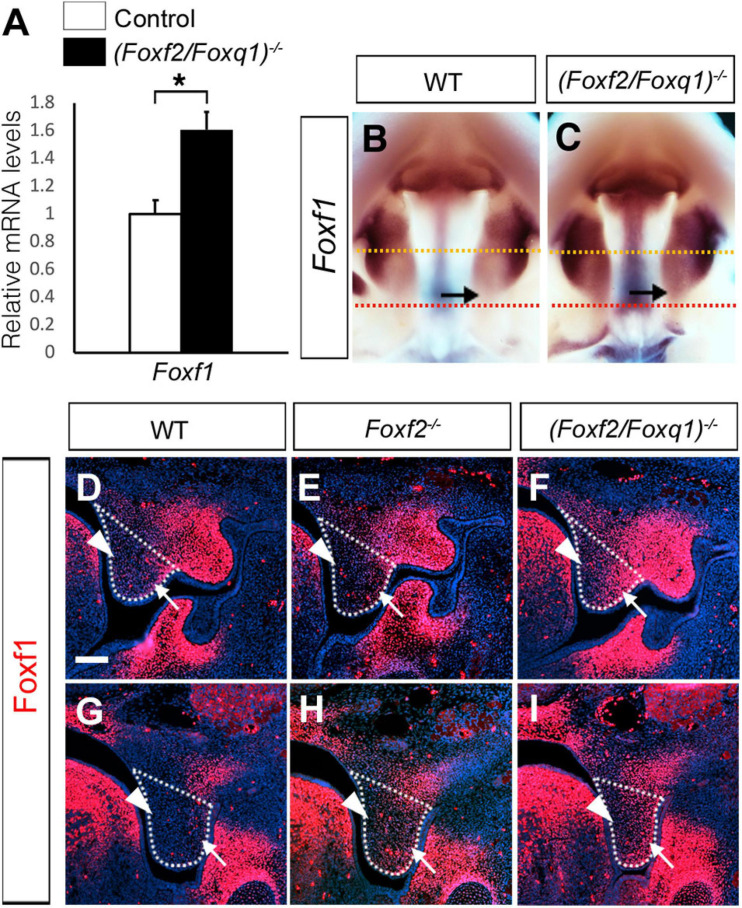
Analysis of expression of *Foxf1* in the developing palatal shelves. **(A)** Real time RT-qPCR analysis of the levels of expression of *Foxf1* mRNAs in E13.5 palatal shelves in control and *(Foxf2/Foxq1)^–/–^* embryos (*n* = 7). *p < 0.05. **(B,C)** Comparison of patterns of expression of *Foxf1* mRNAs in E13.5 wild-type (WT) **(B)** and *(Foxf2/Foxq1)^–/–^* **(C)** mutant embryos. Note that the expression of *Foxf1* mRNAs (detected in purple color) was extended to the posterior region of the palatal shelves in the *(Foxf2/Foxq1)^–/–^* embryos. Arrow points to the posterior region of the palatal shelves. Yellow dashed line marks the approximate position of the plain of frontal sections of different embryos shown in panels **(D–F)**, whereas red dashed line indicates the approximate position of the plain of frontal sections of different embryos shown in panels **(G–I)**. **(D–I)** Immunofluorescent staining showing Foxf1 protein (red) on comparable frontal sections from the middle **(D–F)** and posterior **(G–I)** regions of the palatal shelves in E13.5 wildtype (WT) **(D,G)**, *Foxf2*^–/–^ **(E,H)**, and *(Foxf2/Foxq1)^–/–^* **(F,I)** embryos. The palatal shelf was outlined with white dashed lines. Arrow points to the lateral side whereas arrowhead points to the medial side of the palatal shelves. Scale bar, 100 μm.

We previously generated and analyzed *Foxf1*^*c/c*^; *Foxf2*^*c/c*^; *Wnt1-Cre* compound mutant mice and demonstrated that Foxf1 partly compensated Foxf2 function in palate development (Xu et al., 2016). Another study showed that *Foxf2*^–/–^ mice had increased *Foxf1* expression in the colonic smooth muscle cells and that Foxf2 bound and repressed the *Foxf1* gene promoter in co-transfection assays (Bolte et al., 2015). Our previous RNA-seq analysis did not detect significant changes in *Foxf1* expression in the palatal mesenchyme in *Foxf2*^–/–^ embryos compared with control littermates. However, we found that *Foxf1* expression was significantly increased in the developing palatal shelves in E13.5 *(Foxf2/Foxq1)^–/–^* embryos in comparison with the wildtype littermates by both whole mount *in situ* hybridization and real-time RT-qPCR assays (Figures 7A–C). In the wildtype embryos, expression of *Foxf1* was restricted to the middle portion of palatal shelves at E13.5 (Figure 7B). The expression of *Foxf1* was increased and extended to the posterior region of the palatal shelves in the *(Foxf2/Foxq1)^–/–^* mutant embryos (Figure 7C). We further confirmed these results by immunofluorescent staining using an anti-Foxf1 antibody on frontal sections of wildtype, *Foxf2*^–/–^, and *(Foxf2/Foxq1)^–/–^* embryos (Figures 7D–I). The Foxf1 protein displayed an oral-to-nasal gradient in the developing palatal mesenchyme, and its levels were increased and extended to the nasal side of the middle portion as well as throughout the posterior region of the palatal shelves in E13.5 *(Foxf2/Foxq1)^–/–^* embryos in comparison with the wildtype embryos (Figure 7, compare **F** and **I** with **D** and **G**, respectively). Interestingly, we also consistently detected moderately increased expression of Foxf1 protein in the posterior region of the palatal shelves in *Foxf2*^–/–^ embryos compared with the wildtype embryos (Figure 7, compare **H** with **G**). These results indicate that Foxf2 negatively regulates *Foxf1* gene expression in the developing palatal mesenchyme and that the increased expression of *Foxq1* in the *Foxf2*^–/–^ embryonic palatal mesenchyme partly complemented for Foxf2-mediated regulation of *Foxf1* expression. Altogether, although deletion of *Foxq1* was ultimately insufficient to rescue the cleft palate defects in the *Foxf2*^–/–^ mice, the increased expression of *Foxq1* resulting from the *cis*-regulatory effect of the *Foxf2* mutation affected the regulation of multiple important genes in palate development.

## Discussion

*FOXF2* is located in an evolutionarily conserved gene cluster containing *FOXQ1*, *FOXF2*, and *FOXC1* (Wotton and Shimeld, 2006). Disruption of *FOXF2* is associated with cleft palate and posterior palate defects in humans (Jochumsen et al., 2008; Bu et al., 2015; Seselgyte et al., 2019), making *Foxf2*^–/–^ mutant mouse a good model to study mechanisms of human palate development. Our RNA-seq and *in vivo* expression assays showed that expression of *Foxq1* was the most dramatically upregulated gene in the embryonic palatal mesenchyme in *Foxf2*^–/–^ mouse embryos. Using CRISPR/Cas9 genome editing, we generated (*Foxf2/Foxq1)^+/–^* mice carrying null alleles of *Foxf2* and *Foxq1* in *cis*, and identified a novel *cis-*regulation of *Foxq1* expression by the *Foxf2* mutation during palate development. Our results indicate that Foxq1 affected expression of several previously identified Foxf2-dependent genes in palate development, which needs to be taken into consideration for elucidating the molecular mechanisms involving Foxf2 in regulating palate development.

**FIGURE 8:**
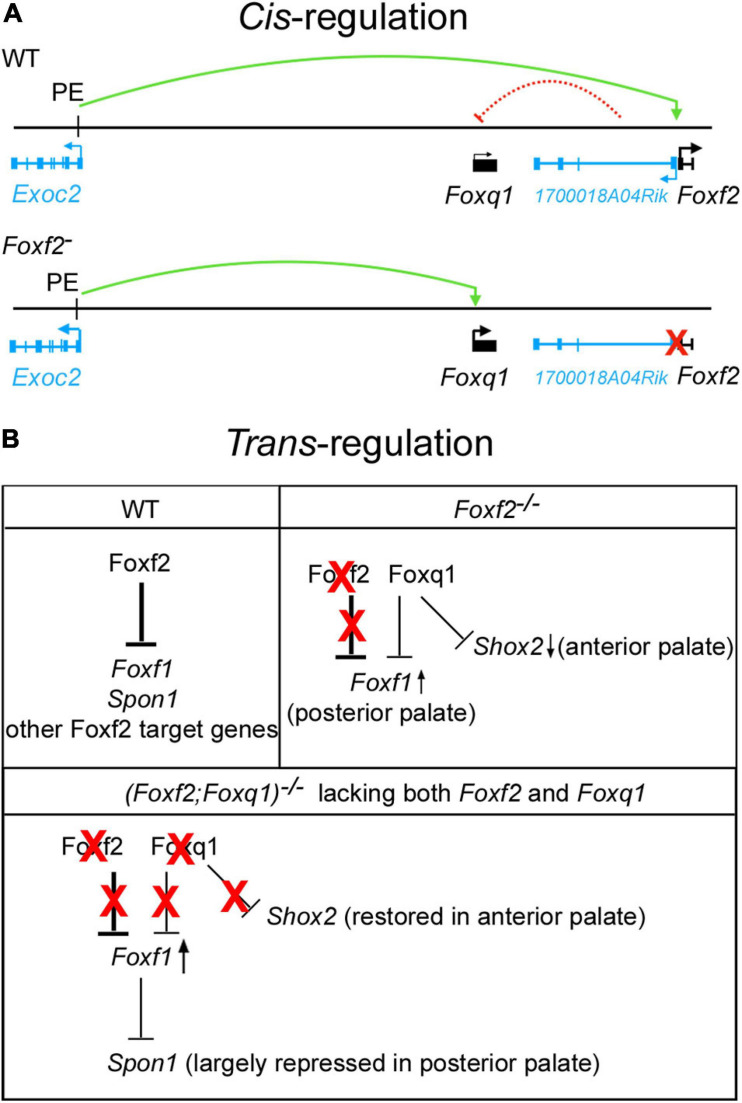
Schematic models of *cis*-regulation of *Foxf2* and *Foxq1* expression and of *trans-*regulation of *Foxf1*, *Shox2*, *Spon1* by Foxf2, and Foxq1 in palate development. **(A)** Model of mechanisms of *cis*-regulation of *Foxq1* and *Foxf2* in palate development. Top panel, in wildtype (WT) embryonic palate, expression of *Foxf2* and the *1700018A04Rik* lncRNA gene is activated through the bidirectional promoter by distal enhancers, including the one identified in intron-1 of the *Exoc2* gene (labeled as PE, for palatal enhancer). Expression of *Foxq1* is not activated and may be repressed by the lncRNA transcript or by transcriptional interference from the *1700018A04Rik* gene in wildtype embryonic palate. The green curved line with an arrow indicates transcriptional activation by the palatal enhancer. The red dashed curved line from the *1700018A04Rik* gene to the *Foxq1* gene indicates a hypothetical *cis-*repression. Bottom panel depicts activation of *Foxq1* gene expression in *Foxf2*^+/–^ and *Foxf2*^–/–^ embryos by the palatal enhancer (PE) due to deletion of the *Foxf2* gene promoter (marked by a red X), which also abolished any *cis*-repressive effect of the *1700018A04Rik* gene on the *Foxq1* allele. **(B)** Models of *trans-*regulation involving Foxf1, Foxf2, and Foxq1. In wildtype embryonic palate, Foxf2 is strongly expressed in the posterior region of palatal shelves and represses expression of *Foxf1*, *Spon1*, and multiple other target genes as previously identified (Xu et al., 2020) in the posterior palatal mesenchyme. In *Foxf2*^–/–^ embryonic palate, Foxf2 protein is absent (marked by red X over “Foxf2”) but *Foxq1* was activated in both the anterior and posterior regions of the palatal shelves. The increased *Foxq1* expression at least partly accounted for the reduction in *Shox2* expression in the anterior palate in *Foxf2*^–/–^ embryos since *Shox2* expression was largely restored in the *(Foxf2/Foxq1)^ –/–^* embryos. *Foxf1* expression was moderately increased in the posterior palate in *Foxf2*^–/–^ embryos and more strongly expressed in the *(Foxf2/Foxq1)^–/–^* embryonic palate, indicating that Foxq1 had a repressive effect on *Foxf1* expression in the *Foxf2*^–/–^ palate. *Spon1* expression was significantly increased in the posterior palate in *Foxf2*^–/–^ embryos but not in the *(Foxf2/Foxq1)^–/–^* embryos, likely due to the increased Foxf1 complementing Foxf2-mediated repression of *Spon1* in the *(Foxf2/Foxq1)^–/–^* embryos. Red X indicates absence of the protein and its regulatory action. Arrow pointing up indicates increased expression, whereas arrow pointing down indicates decreased expression. Note that expression of many Foxf2-dependent genes was similarly altered in the *Foxf2*^–/–^ and *(Foxf2/Foxq1)^–/–^* embryos and are not depicted.

While recent *in vitro* study suggested that FOXF2 binds to the promoter and represses the expression of *FOXQ1* in a breast cancer cell line (Kang et al., 2019), our *in vivo* data demonstrated a *cis*-regulation of *Foxq1* expression by the *Foxf2* gene disruption. *Cis*-regulation of gene expression is primarily mediated by interactions of enhancer and repressor sequences with their target gene promoters. Enhancers can control gene expression in a distance- and orientation-independent manner (Furlong and Levine, 2018). On the other hand, the interactions of enhancers and promoters are controlled by the three-dimensional organization of the genome, which in mammals is organized into a series of TADs that exhibit frequent intra-domain chromatin interactions but relatively rare inter-domain interactions (Dixon et al., 2012; Sexton and Cavalli, 2015). TADs are conserved between different cell types and across species, suggesting that TADs are important for directing enhancer-promoter interactions for controlling spatiotemporal gene expression during animal development (Dixon et al., 2012; Nora et al., 2012). Analysis of the Hi-C data of mouse embryonic stem cells (Bonev et al., 2017; Wang et al., 2018) showed that *Foxf2*, *1700018A04Rik*, *Foxq1*, and *Exoc2* genes are located within the same TAD, whereas *Foxc1* is located in a separate TAD (Figure 3A). We found that, in contrast to the significant increases in expression of *Foxq1* and *Exoc2* mRNAs in the palatal mesenchyme in *Foxf2*^+/–^ and *Foxf2*^–/–^ embryos, expression of *Foxc1* was not significantly altered in the palatal mesenchyme of those mutant embryos compared with wildtype littermates, indicating that the *Foxf2* mutation did not affect the TAD boundary-mediated restriction of enhancer-promoter interactions. The exact molecular mechanism underlying the *cis*-regulatory effect of the *Foxf2* mutation requires further investigation. Previous studies indicated that closely linked gene promoters may compete for the activity of a shared enhancer (Choi and Engel, 1988; Dillon et al., 1997; Lower et al., 2009) and that the sequence composition of core promoters plays a critical role in the specificity of enhancer responsiveness (Merli et al., 1996; Haberle et al., 2019). The ENCODE and Eukaryotic Promoter Database (EPD) projects annotated replicated promoter-enhancer associations between the *Foxf2* gene promoter and three distant enhancers in the genomic region between the *Exoc2* and *Foxq1* genes (Gorkin et al., 2020; Figure 3A), indicating that those enhancers preferentially activated the *Foxf2* gene promoter even though the *Exoc2* and *Foxq1* gene promoters are closer. One of those distant enhancers colocalized with a strong histone H3K27ac peak identified in ChIP-seq analysis of E12.5 mouse palatal tissues (Xu et al., 2019). Another highly enriched H3K27ac peak in the intergenic region between *Exoc2* and *Foxq1* likely marked another active enhancer in the developing palatal mesenchyme (Figure 3A). One possible mechanism is that the distant enhancers that activated *Foxf2* gene expression in the palatal mesenchyme in wildtype embryos interacted with and activated the *Foxq1* gene promoter in *cis* with the *Foxf2* mutant allele (Figure 8A) in *Foxf2*^+/–^ and *Foxf2*^–/–^ embryos. A similar *cis*-regulatory mechanism has been shown to drive increased expression of the *NME4* gene located ∼300 kb away from the α-globin gene cluster in humans when one or more copies of the α-globin gene was deleted (Lower et al., 2009). In addition, the co-transcription of the *1700018A04Rik* lncRNA gene with *Foxf2* may play a role in repressing *Foxq1* gene expression in the developing palatal mesenchyme in wildtype embryos (Figure 8A). A few lncRNAs have been shown to recruit regulatory complexes through RNA-protein interactions to repress nearby genes in *cis* (Maamar et al., 2013; McHugh et al., 2015; Cipriano et al., 2021). Transcription of some lncRNA genes has also been shown to repress expression of a nearby gene in *cis* through transcriptional interference independently of the lncRNA transcript (Houseley et al., 2008; Latos et al., 2012). Whether *1700018A04Rik* plays a detectable role in the transcriptional regulation of *Foxf2* and/or *Foxq1* remains to be determined.

A recent study reported a congenital palate defect with absent uvula, shortened posterior border of the soft palate, and abnormal tonsillar pillar, that cosegregated with a heterozygous missense mutation in *FOXF2* in a large family (Seselgyte et al., 2019). Moreover, submicroscopic 6p25 deletion and/or duplication, including or near the *FOXF2* gene locus, have been associated with syndromic defects including palatal abnormalities in patients (Descipio et al., 2005; Nakane et al., 2013; Seselgyte et al., 2019). Therefore, studying the *cis-*regulation network of this genomic locus will provide valuable insight into the mechanisms of *FOXF2*-related human diseases.

The expression of *Foxq1* was dramatically up-regulated in the palatal mesenchyme cells in *Foxf2*^–/–^ embryos compared with wildtype littermates, but deletion of *Foxq1* didn’t significantly rescue the craniofacial defects or expression of most of the previously identified Foxf2 target genes in the *(Foxf2/Foxq1)^–/–^* embryos. This is possibly due to the relatively low level of expression of *Foxq1* in the *Foxf2*^–/–^ palatal mesenchyme compared with the amount of *Foxf2* mRNAs expressed in the wildtype palatal mesenchyme. On the other hand, previous studies have suggested that FOXF2 and FOXQ1 play opposite roles in controlling epithelial-mesenchymal transition and visceral metastasis in basal-like breast cancer cells (Qiao et al., 2011; Zhang et al., 2011; Cai et al., 2015; Wang et al., 2015; Kang et al., 2019). Other studies showed that FOXF1 and FOXQ1 was able to bind to the same region of the telokin promoter, but they exhibited opposing effects on the promoter activity in colonic smooth muscle cells (Hoggatt et al., 2013). We found that inactivation of *Foxq1* partly restored *Shox2* expression in the anterior palate and reduced the aberrant overexpression of *Spon1* in the posterior palate, but also caused obviously enhanced increase in *Foxf1* expression in both the middle and posterior regions of the palatal mesenchyme, in *(Foxf2/Foxq1)^–/–^* embryos compared with the *Foxf2*^–/–^ and wildtype control embryos (Figure 7). While these data may suggest that Foxq1 acted to repress *Shox2* and *Foxf1* expression while also activating *Spon1* expression in a domain specific manner, the most logical explanation is that the increased expression of *Foxq1* directly or indirectly repressed *Shox2* and *Foxf1* expression in the palatal mesenchyme in the *Foxf2*^–/–^ embryos whereas the more significantly increased expression of *Foxf1* compensated for Foxf2-mediated repression of *Spon1* in the posterior palatal mesenchyme in the *(Foxf2/Foxq1)^–/–^* embryos (Figure 8B). In this scenario, Foxq1 acted as a repressor similar as Foxf2, but also had distinct activity with regards to the regulation of *Shox2* and *Spon1* expression. Furthermore, although *Foxf1* expression was extended into throughout the posterior region of the palatal shelves in the *(Foxf2/Foxq1)^–/–^* embryos, the increased expression of *Foxf1* was insufficient to rescue the cleft palate phenotype or the altered expression of many genes in the posterior palatal mesenchyme that resulted from *Foxf2* disruption, suggesting that Foxf2 has distinct molecular functions in palate development that could not be complemented by either Foxq1 or Foxf1.

In summary, this study demonstrates that disruption of the *Foxf2* gene promoter had a *cis*-regulatory effect on the expression of nearby genes. Our results clearly demonstrate that the increased expression of *Foxq1* in the *Foxf2*^+/–^ and *Foxf2*^–/–^ embryos was due to the *cis*-regulatory effect. Although *(Foxf2/Foxq1)^–/–^* mice exhibited similar cleft palate phenotypes as *Foxf2*^–/–^ mice, Foxq1 exerted a regulatory effect on the expression of several previously identified Foxf2-dependent genes in palate development. We show that *Exoc2*, located about 600 kb upstream of *Foxf2*, was also significantly upregulated in the developing palatal shelves in *Foxf2*^+/–^ embryos and was further significantly upregulated in the *Foxf2*^–/–^ embryos. Whether the increase in *Exoc2* expression in the palatal tissues in *Foxf2*^+/–^ and *Foxf2*^–/–^ embryos was solely due to the *cis*-regulatory effect and whether the altered expression of *Exoc2* contributed significantly to the craniofacial and other developmental defects in the *Foxf2*^–/–^ mice remain to be investigated. Since many gene knockout studies assigned gene function based on promoter-deletion alleles, our finding of a *cis*-regulatory effect of the *Foxf2* mutation calls for caution in interpretation of results and underlying mechanisms from those alleles. Potential *cis-*regulatory effects on neighboring genes should also be taken into consideration when analyzing pathogenicity of human gene deletion variants. In addition, since *Foxf2* mutant mouse studies have shown that multiple tissues and developmental processes, including pericyte development and maturation of the blood-brain barrier, development of the respiratory and digestive organs, in addition to craniofacial and palate tissues, depend on Foxf2 function (reviewed by He et al., 2020), the (*Foxf2/Foxq1)^+/–^* mice provide a valuable resource for understanding the cross-regulation and combinatorial functions of the *Foxf2* and *Foxq1* genes in multiple developmental and disease processes.

## Data Availability Statement

Publicly available datasets were analyzed in this study. This data can be found here: The accession number for the RNA-seq data is GSE67015. The accession number for the palate Foxf2 ChIP-seq data is GSE137585.

## Ethics Statement

The animal study was reviewed and approved by Institutional Animal Care and Use Committee (IACUC) at Cincinnati Children’s Hospital Medical Center.

## Author Contributions

YL and RJ designed the research. JX and HL conducted the experiments and collected the data. JX and RJ wrote the manuscript. All authors analyzed the data, revised the manuscript, and agreed and approved the manuscript.

## Conflict of Interest

The authors declare that the research was conducted in the absence of any commercial or financial relationships that could be construed as a potential conflict of interest.

## Footnotes

1 http://3dgenome.fsm.northwestern.edu

2 https://www.ncbi.nlm.nih.gov/geo/

3 https://genome.ucsc.edu/

4 http://www.informatics.jax.org/snp/rs29587452
