# Supplementary material for: Cis-Repression of Foxq1 Expression Affects Foxf2-Mediated Gene Expression in Palate Development
Source: Front Cell Dev Biol. 2021 Apr 8;9:665109. doi: 10.3389/fcell.2021.665109 (PMC8060495; doi:10.3389/fcell.2021.665109)
Supplement: Supplementary file 1 [file Image_1.pdf]

Supplementary figure for Xu et al., "**Cis-repression of *Foxq1* expression contributes to Foxf2-mediated regulation of palate development**"

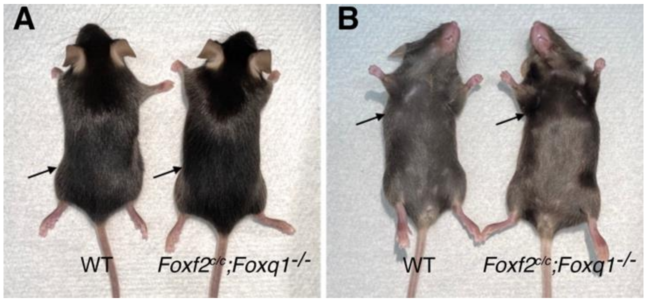

Supplementary Figure 1. *Foxq1* homozygous mutant mice exhibit silky coat.
(A) Dorsal view of wildtype (WT) and *Foxf2*$^{c/c}$*Foxq1*$^{D509/D509}$ homozygous mice.
(B) Ventral view of wildtype (WT) and *Foxf2*$^{c/c}$*Foxq1*$^{D509/D509}$ homozygous mice.
